# Coherently Radiating Periodic Structures for Feeding Concentric Rings Array with Reduced Number of Phase Shifters

**DOI:** 10.3390/s22239528

**Published:** 2022-12-06

**Authors:** Brian Sanchez, Marco A. Panduro, David H. Covarrubias, Alberto Reyna, Elizvan Juárez

**Affiliations:** 1 CICESE Research Center, Electronics and Telecommunications Department, Carretera Ensenada-Tijuana No. 3918, Zona Playitas, Ensenada 22860, Baja California, Mexico; 2 Universidad Autónoma de Tamaulipas, UAMRR-R, Carretera Reynosa-San Fernando, Reynosa 88779, Tamaulipas, Mexico

**Keywords:** phased array, CORPS network, concentric rings, beam-scanning, side lobe level

## Abstract

This paper presents the application of CORPS (coherently radiating periodic structures) for feeding CRA (concentric rings array) with a reduced number of phase shifters. The proposed design technique for the structure of concentric rings provides a better scanning capability with respect to other existing configurations. This design technique utilizes 2 × 3 or 4 × 7 CORPS networks depending on the configuration or the number of antenna elements in the phased array system. These CORPS networks are set strategically in the feeding network to provide several advantages with respect to others in the scanning capability and the reduction of the number of phase shifters of the array system. The contribution of this paper is the full antenna system design of phased CRA for analyzing scanning and the reduction of phase shifters. The proposed phased array reduces the number of phase shifter devices in CRA for a scanning range of ±25° in the elevation plane. Differential evolution (DE) was applied to optimize the amplitudes of the proposed system. Several design cases were analyzed using full-wave simulation results to verify the phased array model and to take mutual coupling into account. Full-wave simulation results provide radiation patterns with low SLL in all scanning directions. The proposed phased array was validated by experimental measurements of the full antenna system prototype.

## 1. Introduction

There is great interest about minimizing the number of electronic devices used in the feeding network of the antenna system while maximizing the radiation pattern quality [1]. Two of the main components of phased array systems are the array architecture and the beam-forming network (BFN). The first one determines the spatial distribution of each antenna element, and the BFN is a crucial component for feeding each antenna element of the system with adequate amplitudes and phases. An adequate BFN design allows us to generate radiation patterns with desirable properties such as a wide range of beam-scanning and low side lobe level (SLL) [2,3].

The complexity and cost of phased array systems depend strongly on the BFN. This complexity is high for a high number of electronic devices (phase shifters, amplifiers, switches, etc.) [4]. It is crucial to find out different antenna arrays geometries and BFN configurations for minimizing the number of electronic devices [5,6,7] in applications of wide range scanning and low SLL. There are several recent techniques in the state of art such as: checkered networks [8], minimal redundancy [9], hybrid beamforming [10], rotated elements [11], directional modulation [12], and coherently radiating periodic structures (CORPS) [13], among others.

The techniques based on CORPS have been studied for reducing the number of phase shifters in the BFN. Several CORPS network configurations have been analyzed yielding a better scanning capability with respect to other BFN configurations [14,15,16,17,18,19,20,21,22].

The previously mentioned works have provided several design techniques for feeding different antenna array structures such as linear, planar, and concentric rings. In this sense, new design methodologies are needed to minimize the complexity of the feeding system of the antenna array. All these methodologies can set more design options for emerging systems based on antenna arrays.

Our manuscript illustrates the use of CORPS to feed antenna array systems with a geometry of concentric rings by using a minimum quantity of phase shifters. The phased antenna system using the proposed CRA uses cophasal excitation and provides an interesting option for feeding the antenna elements and diminishing the quantity of phase devices. This antenna system design using the geometry of CRA can achieve a wide scanning range. In this case, 2 × 3 or 4 × 7 CORPS networks are utilized in the proposed CRA to provide the required phase distribution. The proposed design methodology using these CORPS blocks achieves some benefits, such as a wide scanning of the main beam and the simplification of the BFN by diminishing the quantity of phase shifters of the antenna system. The differences with respect to our previous work published in [22] are the reduction of phase shifters for a configuration based on arrays of concentric rings and the analysis of the radiation performance for different configurations.

The contribution of this paper is the full antenna system design of phased CRA for analyzing scanning and the reduction of phase shifters. The proposed phased array reduces the number of phase shifter devices in CRA for a scanning range of ±25° in the elevation plane. Differential evolution (DE) [23] was applied to optimize the amplitudes of the proposed system. Several design cases are analyzed using different numbers of antenna elements with full-wave simulation results (using CST Microwave Studio) to verify the phased array model and to take mutual coupling into account. Full-wave simulation results provide radiation patterns with low SLL in all scanning directions. The proposed phased array was validated by experimental measurements of the full antenna system prototype.

## 2. Phased Array Model

### 2.1. Concentric Rings Array

The geometry of concentric rings is considered for the phased array model. We have selected this geometry because the behavior of the required cophasal excitation for this structure facilitates the application of CORPS.

**Figure 1:**
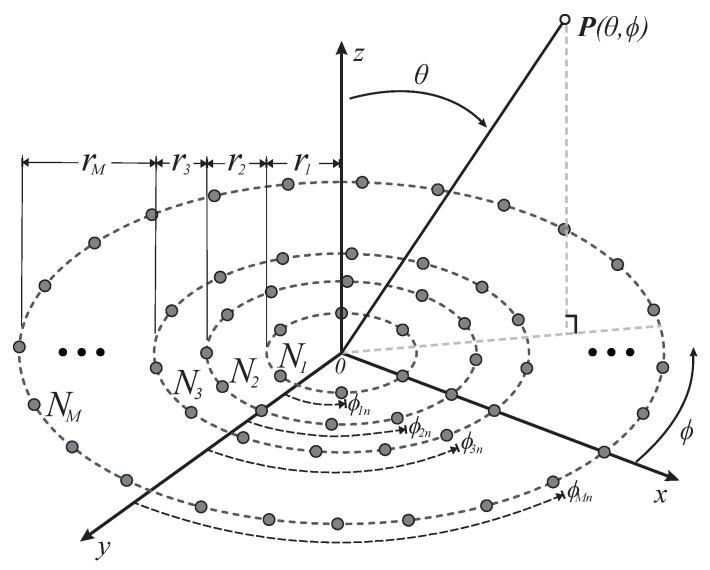
Geometry of the concentric rings array.

Basically, this array geometry incorporates multiple rings with antenna elements in a circular layout, sharing a common center where each ring has its own radius [21]. This concentric ring geometry considers *M* rings with $N_{m}$ antenna elements on the *x*-*y* plane, as shown in Figure 1. Thus, the array factor expression considering this structure can be written as follows [21,24]:

(1) $$\mathrm{AF}\left(\theta ,\;\phi \right)=\overset{M}{\underset{m=1}{\sum }}\overset{N_{m}}{\underset{i=1}{\sum }}W_{m}e^{{\mathrm{jkr}}_{m}\left(\lambda \right)\left[\sin\theta \cos\left(\phi -\phi_{\mathrm{mi}}\right){+\sin\theta }_{0}\cos\left(\phi_{0}-\phi_{\mathrm{mi}}\right)\right]}$$

Where *M* is the number of rings, *N_m_* is the number of elements in ring *m*, *W_m_* is the excitation current of elements on *m*th ring, k is the angular wavenumber with *λ* as the wavelength, *r_m_*(*λ*) is the radius of each circular ring with inter-element spacing between rings of *d_m_*; *θ* ∈ [0, π] and ϕ ∈ [0, 2π] are the elevation and azimuth angles (from the positive *z-axis* and from the positive *x-axis*), respectively. The maximum radiation direction is defined by *θ*_0_ and *ϕ*_0_. The element angular separation *ϕ_mi_* from the positive *x*-axis in each ring can be calculated as *ϕ_mi_* = 2π(*i* − 1)/*N_m_*.

We analyzed the impact of the application of CORPS for reducing the number of phase shifter devices in this geometry of concentric rings. Two design configurations were proposed in order to take advantage of the CORPS properties for providing the required cophasal excitation for this structure and to consider the scanning possibilities.

**Figure 2:**
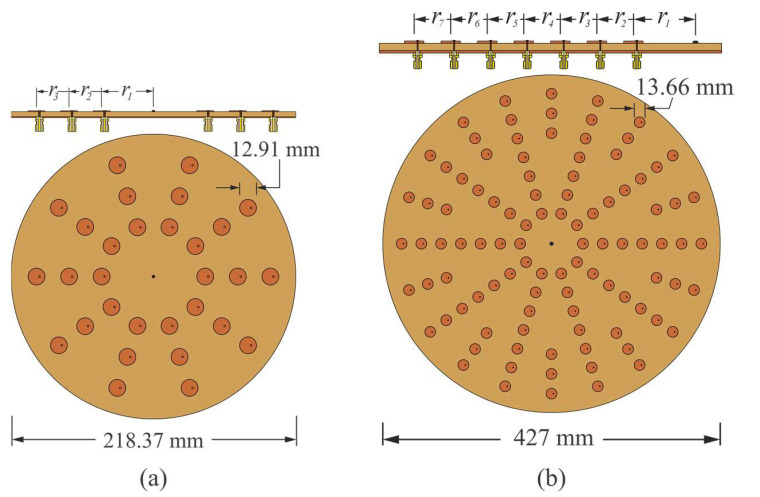
Array configurations: (**a**) Configuration 1 of three rings with 30 antenna elements, (**b**) Configuration 2 of seven rings with 100 antenna elements, both at 6 GHz.

Configuration 1, shown in Figure 2a, considers three rings with 10 antenna elements per ring. This kind of array configuration permits the control of blocks of three antenna elements. Each block had the same angular separation. Then, this configuration can utilize CN2×3 (2 × 3 CORPS networks) and take advantage of the phase interpolation property of CORPS networks [22] for generating the required cophasal excitation.

The array configuration assumes the distance between antenna elements of the same ring to be 0.5λ, trying to avoid the effects of mutual coupling. Then, the corresponding radii for configuration 1 are: $r_{1}=0.7958\lambda$, $r_{2\;}=1.2958\lambda ,$ and $r_{3}=1.7958\lambda$. Figure 2a shows the antenna array using a circular patch with a central frequency of 6 GHz, diameter of 12.91 mm, $p^{\prime }$ = 2.07 [25], the FR4 substrate with a thickness of *h* = 1.6 mm, relative permittivity $e_{r}=4.2$, and tangent loss *δ = 0.025*.

The other interesting array configuration is illustrated in Figure 2b. As shown in Figure 2b, configuration 2 uses 100 antenna elements distributed over seven rings. This configuration permits the control of blocks of seven antenna elements and blocks of three elements. Therefore, the array configuration can use CN4×7 (4 × 7 CORPS networks) and CN2×3 in order to generate the cophasal excitation for beam-scanning and reducing the number of phase shifter devices. As in configuration 1, the array configuration assumes the distance between antenna elements to be 0.5λ, having the next radii values: $r_{1}=0.7958\lambda$, $r_{2}=1.2958\lambda$, $r_{3}=1.7958\lambda$, $r_{4}=2.2958\lambda$, $r_{5}=2.7958\lambda$, $r_{6}=3.2958\lambda ,$ and $r_{7}=3.7958\lambda$.

### 2.2. CORPS Networks

The feeding networks are based on CORPS to be used as a beam-forming network of the concentric rings array. The CORPS networks take advantage of the wave propagation, splitting (S), and recombining (R) the waves or signals in basic nodes [14,15]. We applied the CORPS networks for taking advantage of the phase interpolation property of a CORPS network of one layer.

**Figure 3:**
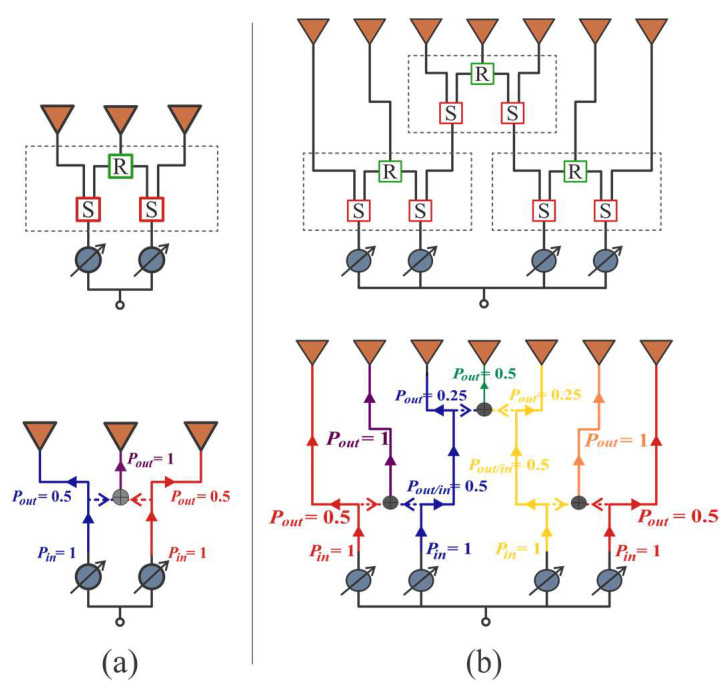
CORPS networks. (**a**) CN2×3, which uses 2 splitting uses and 1 recombining node, (**b**) CN4×7, which uses 6 splitting nodes and 3 recombining nodes.

The C-BFN configurations were designed according to the CRA model structure. As commented previously, the CRA of configuration 1 utilizes CN2×3 and the CRA of the configuration 2 uses CN4×7 and CN2×3 in order to generate the cophasal excitation for beam-scanning. The CN2×3 uses 2 splitting nodes and 1 recombining node as shown in Figure 3a, and the CN4×7 is designed by interconnecting several CN2×3 [22] (Figure 3a). Unlike a CORPS conventional network (which has 15 splitting and 12 recombining nodes), this configuration uses only 6 splitting and 3 recombining nodes [22]. The theoretical power distribution is illustrated in Figure 3b. 

**Figure 4:**
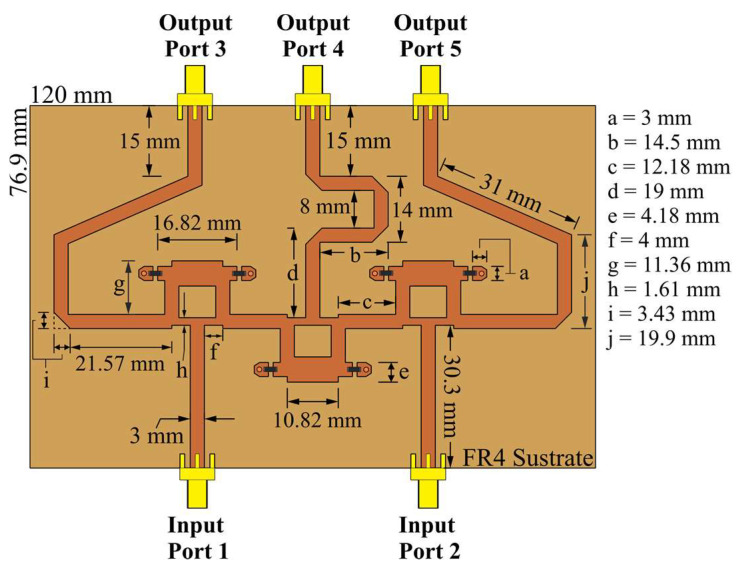
CN2×3, which uses 3 Gysel power dividers at 6 GHz in CST Studio Suite.

**Figure 5:**
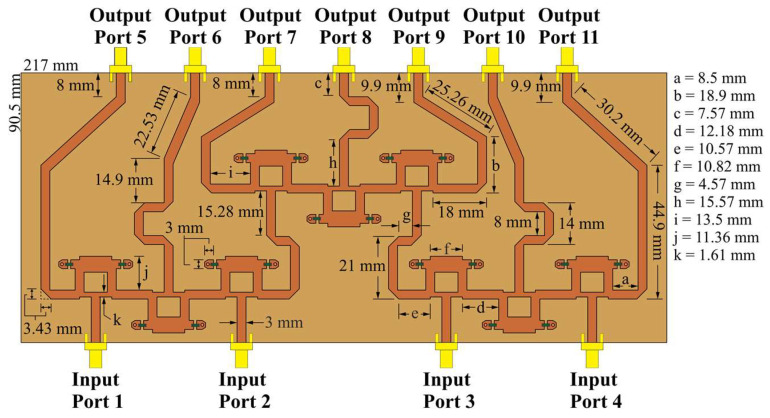
CN4×7, which uses 9 Gysel power dividers at 6 GHz in CST Studio Suite.

Gysel power dividers [26] can perform the practical functions of the splitting and recombining nodes. Figure 4 and Figure 5 illustrate the CN2×3 and CN4×7 using Gysel dividers. These configurations incorporate the FR4 substrate with a thickness of 1.6 mm, relative permittivity $e_{r}=4.2$, and tangent loss *δ = 0.025*. Gysel power dividers include chip resistors of 50 Ohms (FC0603-surface mount) and SMA connectors at each input and output port. 

**Figure 6:**
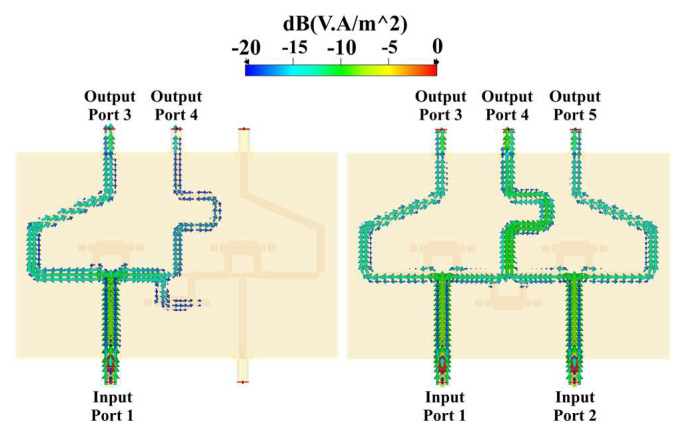
Power flow through the CN2×3.

**Figure 7:**
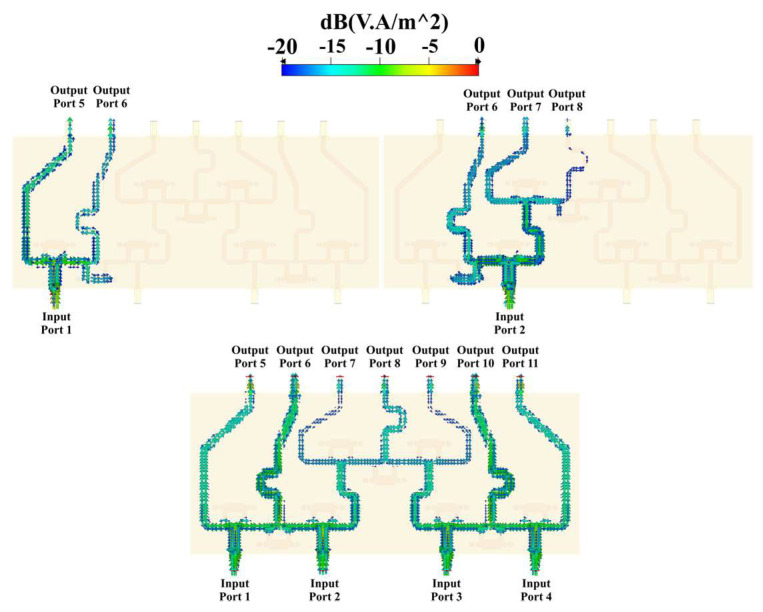
Power flow through the CN4×7.

The power flow through the networks can be simulated. CN2×3 includes a combination of 3 Gysel power dividers that produce power attenuations (≈−3 dB per divider). Figure 6 shows the simulated power flow of the CN2×3 when the input ports are fed separately and simultaneously by a 6 GHz signal with the same phase value and a unitary amplitude. The Gysel power divider acts as a splitting or combining node of the network. In addition to this, Figure 7 shows a combination of 9 Gysel power dividers, which correspond to 3 CN2×3 interconnected. Thus, this network reduces until 66%, the required Gysel power dividers with respect to the conventional CORPS network. Please note that the performance details of these networks (CN4×7 and CN2×3) such as reflection and transmission coefficients are explained and found in the references [20,22].

Figure 7 shows the simulated power flow of the CN4×7 when the input ports are fed separately and simultaneously by a 6 GHz signal with the same phase value and a unitary amplitude. This configuration has a symmetric performance between the first two input ports and the last two input ports of the network, in which the power flow, attenuation, and phase shift are the same at the corresponding output ports. The maximum phase shift of the signal is found in the center. The phase shift can be adjusted at the ends of the network, generating longer lines.

**Figure 8:**
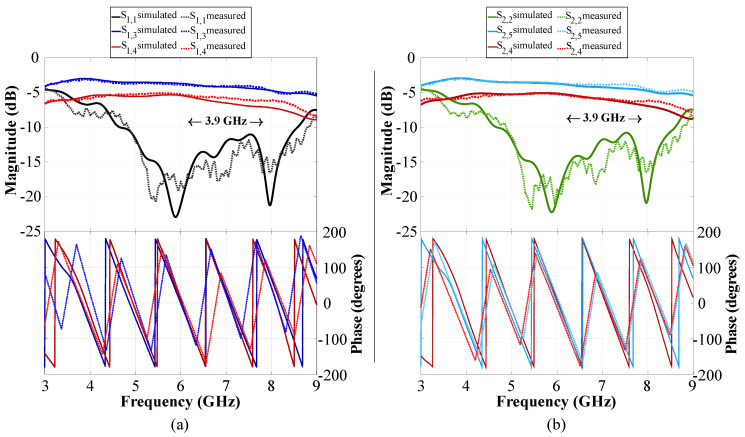
S-parameters of the CN2×3: (**a**) input 1 to outputs 3 and 4, (**b**) input 2 to outputs 4 and 5.

**Figure 9:**
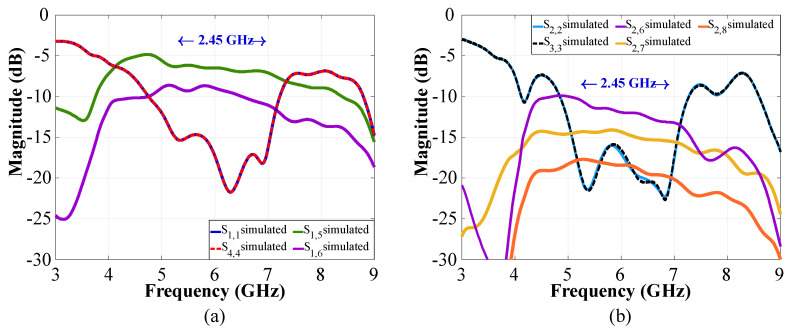
S-parameters of the CN4×7: (**a**) inputs 1 and 4, (**b**) inputs 2 and 3.

The behavior of CN2×3 and CN4×7 can be studied using simulations and measurements. The S-parameters of CN2×3 and CN4×7 are illustrated in Figure 8 and Figure 9 in terms of reflection and transmission coefficients and phase versus frequency. The CN2×3 shows a bandwidth of 3.9 GHz (as shown in Figure 8) with reflection coefficients of less than −20 dB and −18 dB in the simulated and measured results at 6 GHz, respectively. The transmission values are approximately −3.75 dB in the ports 3 and 5 and approximately −5.35 dB in the port 4. 

Figure 9 shows a reflection coefficient of −20 dB for the CN4×7 considering the input ports 1 and 4, and −15 dB for the ports 2 and 3 (6 GHz). This network presents a bandwidth of 2.45 GHz. The transmission values obtained are $S_{1,5–4,11}=-7.52\;\mathrm{dB}$ and $S_{1,6–4,10}=-10.4\;\mathrm{dB}$ for the input ports 1 and 4, and of approximately $S_{2,6–3,10}=-11.1\;\mathrm{dB}$, $S_{2,7–4,9}=-12\;\mathrm{dB},$ and $S_{2,8–4,8}=-15.3\;\mathrm{dB}$ for the ports 2 and 3.

### 2.3. Proposed Model for Phased Antenna Arrays

Each phased antenna array system (PAAS) was developed by the interconnection of the CORPS-BFN configuration with its corresponding CRA case. These interconnections are realized from the output of the network to each antenna element in the different rings of the array and the different array inputs are arranged or set as linear subsets. This simplifies the phased array system by a feeding network that uses a smaller number of phase shifters.

**Figure 10:**
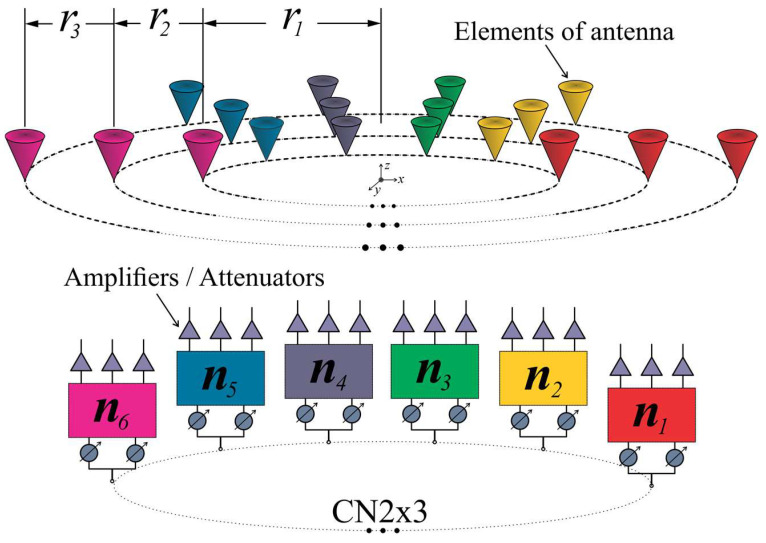
Schematic diagram for the phased antenna array system of configuration 1, including CN2×3.

Figure 10 illustrates a schematic diagram for the PAAS of the CRA configuration 1, including 10 CN2×3. A layer of attenuators/amplifiers was set at the output of the feeding networks. These values of amplitude excitation can be optimized in order to yield radiation pattern characteristics with a low SLL for each main beam direction. These values of amplitude excitation are considered fixed, i.e., the same value of attenuation is used in each antenna element for each scanning direction.

As an example, Table 1 shows the different phase values generated by the CN2×3 at $\theta_{0\;}=25{}^{\circ}$ in the cut of ϕ=0°. The computed phase value is illustrated for each antenna element of the CRA. Because of symmetry of the CRA geometry, the CN2×3 (the networks 2–10, 3–9, 4–8, and 5–7) generate the same phase values for each CN2×3 pair at the output of the feeding network. It is important to note that the input phase difference above 180° could generate power reflections at the input ports due the passive elements of the network. Therefore, phase differences above 180° (between input ports of CN2×3) must be absolutely avoided. This situation is not presented in this arrangement of antenna elements and CN2×3 for the CRA of configuration 1. This configuration provides a scanning range ±25°. Please, take note that this scanning performance is reached by controlling 30 elements of the CRA for this configuration with 20 phase shifters. There was a reduction of 33% in the number of phase shifters for phased arrays in a geometry of CRA.

**Figure 11:**
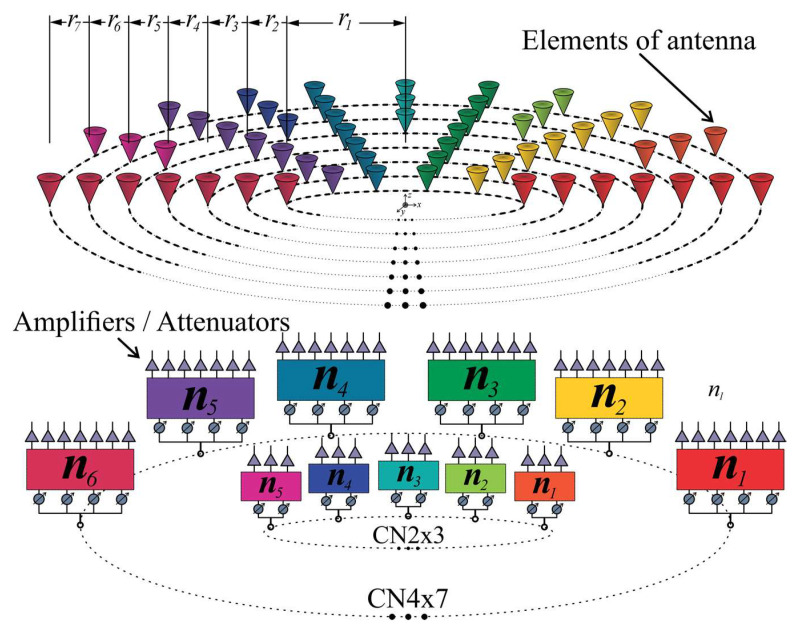
Schematic diagram for the phased antenna array of configuration 2, including CN4×7 and CN2×3.

The schematic diagram for the PAAS of the CRA configuration 2 is shown in Figure 11. This configuration includes 10 CN2×3 and 10 CN4×7. Although 70 antenna elements could be controlled by the 10 CN4×7, 30 antenna elements are controlled by 10 CN2×3. These 30 elements set in 10 subarrays of 3 elements are added in order to provide better radiation characteristics. A layer of attenuators/amplifiers is set at the output of the feeding networks and these values of amplitude excitation are considered fixed, as previous configuration. Therefore, this configuration provides a scanning range ±25° by controlling 100 elements of the CRA for this configuration with 60 phase shifters. There was a reduction of 40% in the number of phase shifters.

Table 2 illustrates the different phase values generated by the 10 CN2×3 and the 10 CN4×7 at $\theta_{0}=25{}^{\circ}$ in the cut of ϕ=0°. As in the previous configuration, the CN4×7 (networks 2–10, 3–9, 4–8, and 5–7) and the CN2×3 (networks 1–10, 2–9, 3–8, 4–7, and 5–6) generate the same phase values (for each networks pair mentioned previously) at the output of the feeding network. As shown in Table 2, the phase differences above 180° between input ports are avoided.

## 3. Experimental Results

Simulation and experimental results were obtained for the PAAS of the CRA configurations. The full antenna system for the PAAS of the CRA configuration 1 was full wave simulated, fabricated, and measured for evaluating its performance. The PAAS of CRA configuration 2 was only evaluated by using full wave simulations due to the high complexity.

**Figure 12:**
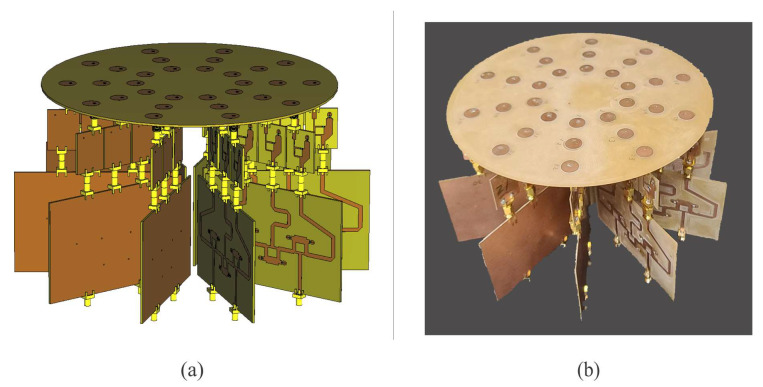
Full system design for the CRA configuration 1: (**a**) design and (**b**) prototype.

Figure 12a shows the full system design for the PAAS of the CRA configuration 1. The prototype was fabricated using the array design characteristics mentioned in Section 2.1 (Figure 12b). The design and the prototype illustrate the feeding network based on CN2×3, the attenuators, and the antenna elements of the CRA. All was constructed on FR4 substrate. More details are given in the next sections.

**Figure 13:**
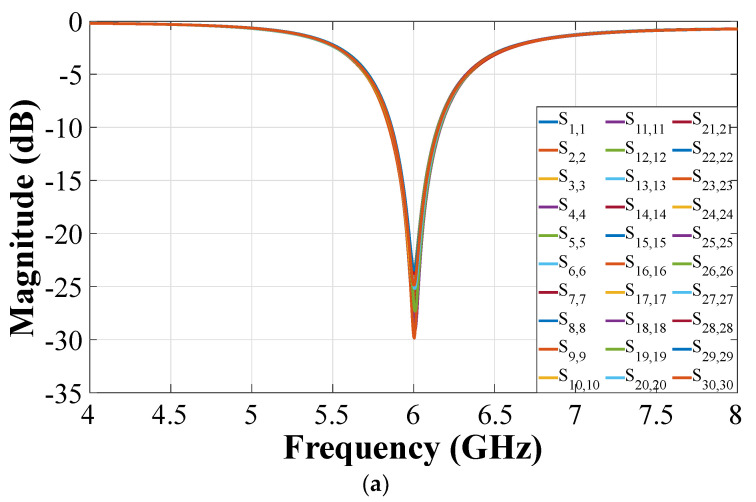

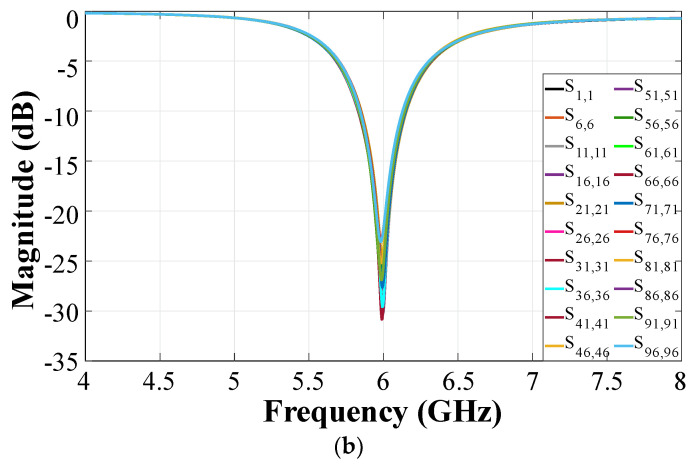
Active reflection coefficients for the CRA of (**a**) configuration 1 and (**b**) configuration 2 at *θ*_0_ = 25° for a frequency of 6 GHz.

Figure 13 illustrates the active reflection coefficients for the CRA of configuration 1 and configuration 2 at $\theta_{0}=25{}^{\circ}$. Every direction of the scanning range was examined, as this was the furthest scanning direction and the case of worst performance for active reflection coefficients. The reflection coefficients of all antenna elements remained below −10 dB at the design frequency of 6 GHz. The antenna elements show a good matching performance for the frequency of interest.

The amplitude excitations were optimized, intending to find radiation patterns with low SLL in all scanning directions for the PAAS of the two CRA configurations. The optimization process was made using a metaheuristic algorithm based in the DE [23]. The principal aim of this algorithm was to determine and select, from a random vector, the best combination of amplitudes for each CRA configuration. This algorithm has been applied extensively and successfully in the array design. Therefore, the details of this optimization algorithm can be found in [23]. We seek with this optimization to represent the use of a set of passive attenuators (due to the low cost of the fabrication). The DE algorithm was run with an initial population of 100 and a number of generations of 1000 iterations. Furthermore, the optimization process considers the next cost–function (*CF*), which evaluates the radiation parameters of SLL and Directivity (D):

(2) $$\mathrm{CF}=W_{1}\mathrm{SLL}\left(\theta \right)-W_{2}/D\left(\theta \right)\;$$

where *W*_1_ and *W*_2_ are weighting factors. The values of W_1_ and W_2_ can be set to give more importance to reduce the SLL or to improve directivity. Then, the values of SLL and directivity (used in the *CF*) are compared and evaluated by the DE algorithm trying to minimize the *CF* in Equation (2). The radiation pattern is considered to be scanned in the elevation plane in a range of *θ*_0_ = ±25°. Steps of 1° are considered to evaluate all the scanning range and the worst radiation characteristics (SLL and D) are obtained for the farthest direction. Table 3 presents the vectors of different amplitudes computed with DE algorithm for each CRA configuration.

**Figure 14:**
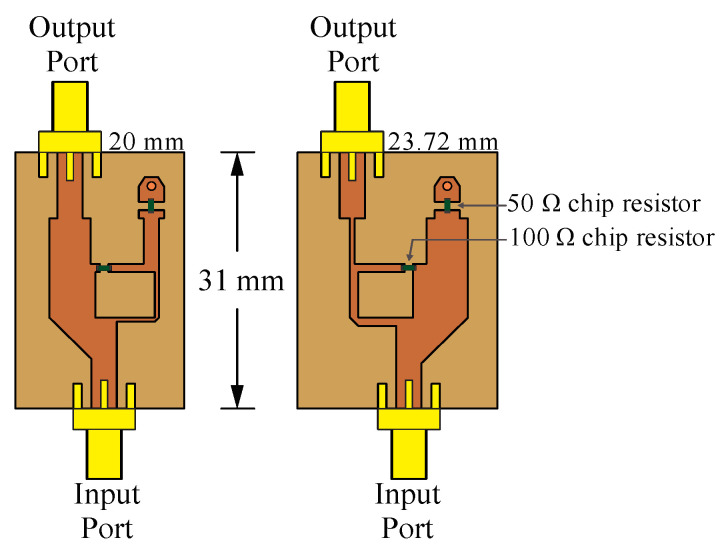
Design of attenuators in CST Studio Suite at 6 GHz.

Each attenuator of the prototype illustrated in Figure 14 was realized considering unequal Wilkinson power divider [27]. The split tee power divider consists in a common power divider with an isolated port. By this way, the output port has an attenuated signal from input [27]. The attenuation level is controlled by the different transmission lines width. Figure 14 illustrates the design of attenuators in CST Studio Suite with the split tee power dividers and (surface mount) resistors of 50 and 100 Ohms for attenuation levels of −1.86 dB and −8.13 dB of attenuation.

All the attenuators were full wave simulated and measured to be integrated with the feeding network of CN2×3, i.e., the full system design for the PAAS of the CRA configuration 1 shown in Figure 12b. All the attenuation coefficients for the full system design (the simulation model in CST and the prototype illustrated in Figure 12a) are shown in Table 4. The attenuation values are given in dB.

Experimental measurements of the radiation pattern for the prototype of the PAAS for configuration 1 (Figure 12b) were performed in a far field anechoic chamber. It is important to note that this phased array design generates (or considers) a cophasal excitation across the antenna elements. More work and study could be done in the phase control in order to generate an optimal phase distribution, but this was outside the aim of our paper. In our case, the measurement system generates the phase shifts required at the input ports to generate a cophasal excitation across the CRA. These delays or transmission lines can be replaced by electronic phase shifters without change in the radiation pattern performance [6].

**Figure 15:**
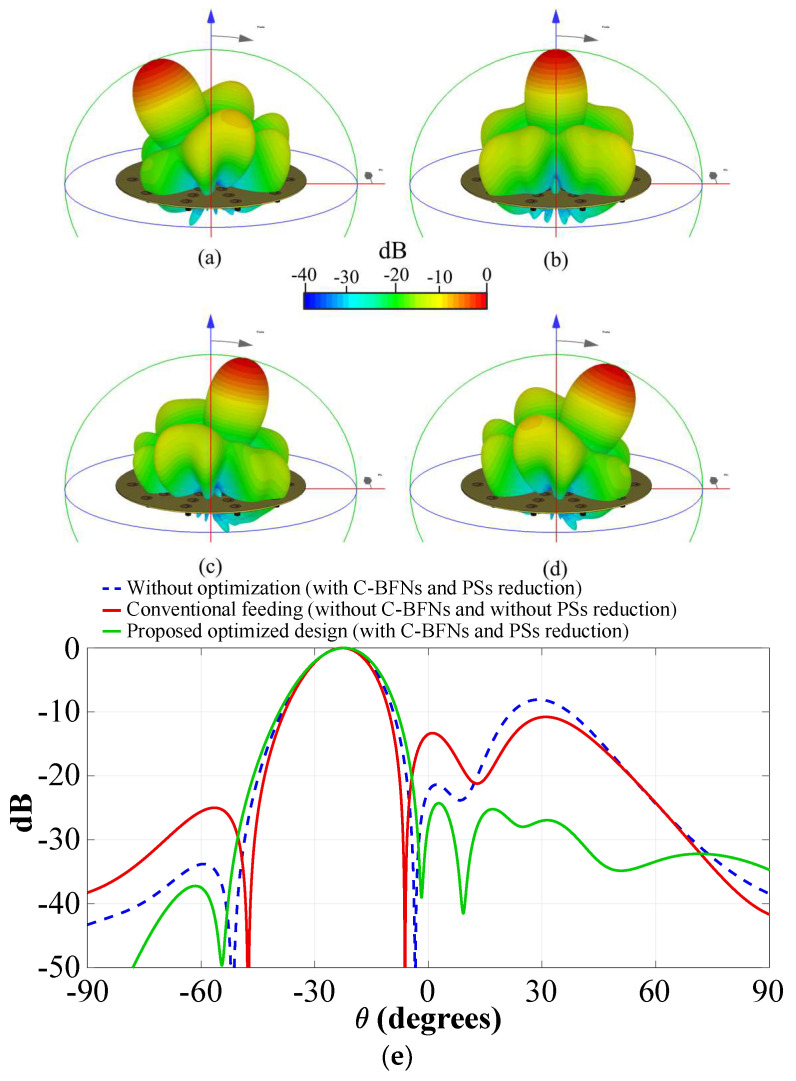

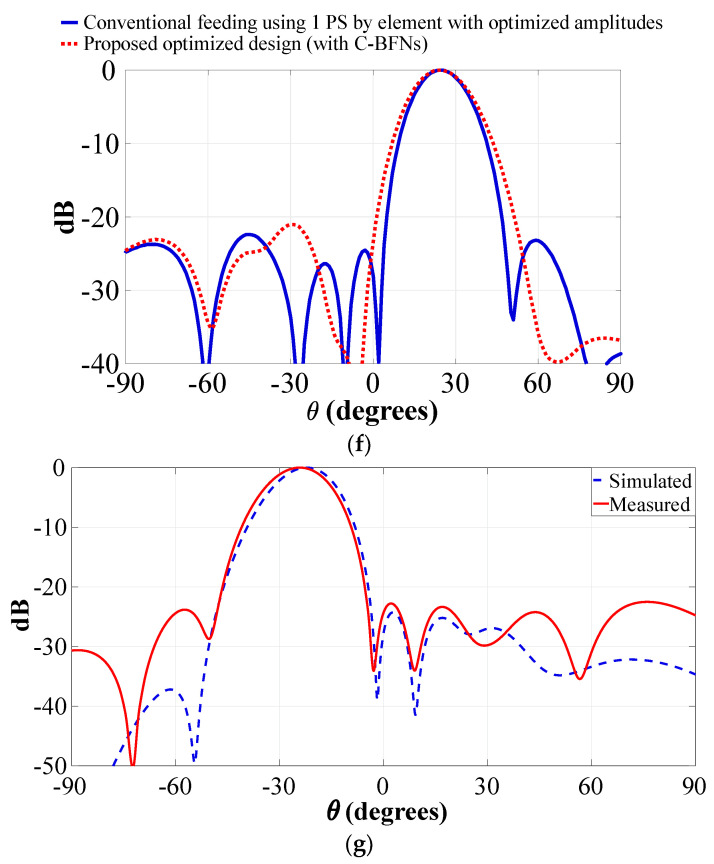
Radiation pattern obtained for the system of the CRA configuration 1 (Figure 12) by full-wave simulations (CST) (**a**) *θ*_0_ = −25°, (**b**) *θ*_0_ = 0°, (**c**) *θ*_0_ = 15°, (**d**) *θ*_0_ = 25°, (**e**) comparison of the radiation pattern with respect to conventional techniques, (**f**) comparison of the radiation pattern with respect to the case of using 1 PS by element (optimized amplitudes), and (**g**) experimental measurements.

Figure 15 shows the radiation pattern obtained by full wave simulations (in CST) and experimental measurements, (a) *θ*_0_ = −25°, (b) *θ*_0_ = 0°, (c) *θ*_0_ = 15°, and (d) *θ*_0_ = 25°, for the system of the CRA configuration 1 (shown in Figure 12). These results illustrate that the design of the phased CRA (using the proposed technique) provides a reduction of phase shifters (33%) generating a radiation pattern with low SLL. This SLL performance remained in all scanning ranges. The radiation pattern obtained by the proposed design was compared (in Figure 15e) with respect to the case without amplitude excitations optimization and the uniform conventional CRA. The optimization of the amplitude coefficients generates a radiation pattern with a SLL value of −24 dB and a reduction of ≈11 dB in the SLL with respect to the other techniques. Furthermore, the radiation pattern obtained by the proposed optimized design with the reduction of phase shifters is compared with respect to the case of using 1 phase shifter by elements with optimized amplitudes (Figure 15f). Evidently, as shown in Figure 15f, there is a cost to having the reduction of phase shifters (1.5 dB of SLL with respect to the optimal case using 1 PS per element). The phased antenna system based on CRA configuration 1 was validated by experimental measurements as illustrated in Figure 15g). The radiation pattern results obtained by experimental measurements agree with the simulation results with a slight deviation in the SLL performance (−22.5 dB).

**Figure 16:**
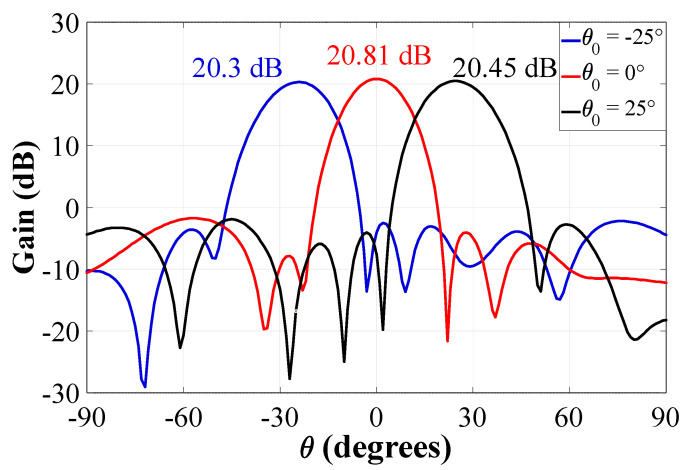
Gain values of the CRA configuration 1 considering beam-scanning.

Furthermore, the scan-loss of the CRA configuration 1 is shown in Figure 16. The gain losses of the radiation pattern with respect to the natural response at the direction of *θ*_0_ = 0° (without beam steering) are approximately −0.51 dB and −0.36 dB at *θ*_0_ = −25° and *θ*_0_ =25°, respectively. The results demonstrate low losses in the gain values of the radiation pattern in the entire scan range, even when any phase shift is used (from *θ*_0_ = 0° to *θ*_0_ = ±25°).

**Figure 17:**
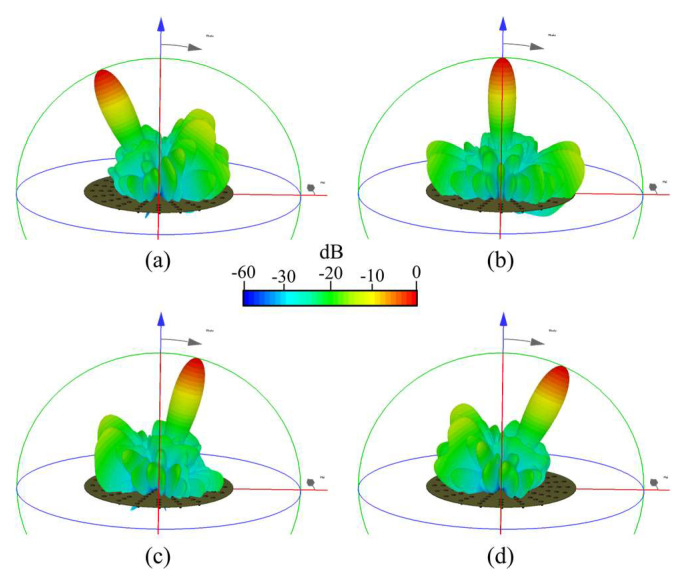

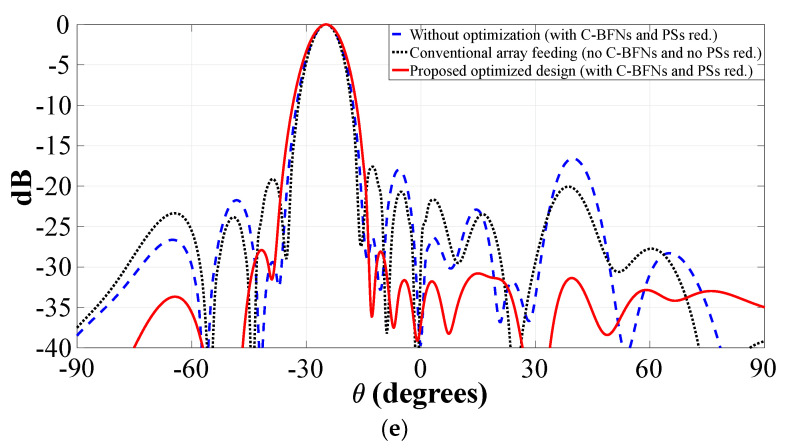
Radiation pattern obtained for the system of the CRA configuration 2 by full-wave simulations (CST) (**a**) *θ*_0_ = −25°, (**b**) *θ*_0_ = 0°, (**c**) *θ*_0_ = 15°, (**d**) *θ*_0_ = 25°, (**e**) comparison with respect to conventional techniques.

Figure 17 illustrates the radiation pattern obtained by full-wave simulations for the system of the CRA configuration 2 at (a) *θ*_0_ = −25°, (b) *θ*_0_ = 0°, (c) *θ*_0_ = 15°, and (d) *θ*_0_ = 25°, and a comparison is shown in Figure 17e (with respect to the uniform conventional CRA and without optimization). As shown in Figure 17, the radiation pattern obtained by the proposed design for this CRA configuration provides a SLL value of −27.9 dB, a significant reduction of SLL (≈11.2 dB) with respect to the conventional techniques. This SLL performance was reached by a reduction of 40% in the number of phase shifters in all scanning ranges.

Table 5 illustrates a comparative analysis of the proposed design for PAAS of the CRA configurations with respect to other state of art techniques (for designing phased arrays in the geometry of CRA). This comparative analysis is made in terms of the SLL, reduction of phase shifters, number of antenna elements, and scanning range. The proposed design for the CRA configurations provides a reduction in phase shifters (of 33% for configuration 1 and 40% for configuration 2) for a scanning range of ±25° with a peak SLL of −24 dB (full-wave simulations) (−22.5 dB obtained with measurements) for configuration 1 and −27.9 dB (full-wave simulations) for configuration 2.

## 4. Conclusions

The proposed design technique for the structure of concentric rings provided a better scanning capability with respect to other existing configurations. CORPS networks were set strategically in the feeding network to provide several advantages with respect to others in the scanning capability and the reduction of the number of phase shifters of the array system. The proposed phased array reduced 33% and 40% of the phase shifters devices for CRA configuration 1 and configuration 2, respectively. DE was applied to optimize the amplitudes of the proposed system for both CRA configurations. 

The proposed design for the CRA configurations provided a reduction in phase shifters for a scanning range of ±25° with a peak SLL of −24 dB (full-wave simulations) (−22.5 dB obtained with measurements) for CRA configuration 1 and −27.9 dB (full-wave simulations) for CRA configuration 2. The proposed phased array achieves a good design compromise with respect to other techniques in the literature.

## Figures and Tables

**Table 1:** Phases values in each element of antenna grouped in each output port for CN2×3 of CRA of configuration 1.

CN2×3 No.	Element of Antenna	Phase Value (Rad)
1	1, 11, 21	−2.11, −3.44, −4.77
2; 10	2, 12, 22; 10, 20, 30	−1.71, −2.78, −3.86
3; 9	3, 13, 23; 9, 19, 29	−0.65, −1.1, −1.47
4; 8	4, 14, 24; 8, 18, 28	0.65, 1.1, 1.47
5; 7	5, 15, 25; 7, 17, 27	1.71, 2.78, 3.86
6	6, 16, 26	2.11, 3.44, 4.77

**Table 2:** Phases values in each element of antenna grouped in each output port for configuration 2.

Network No.	Antenna Element	Phase Value (Rad)
CN4×7		
1	1, 11, 21, 31, 41, 61, 81	−2.11, −3.44, −4.77, −6.1, −7.42, −8.75, −10.08
2; 10	2, 12, 22, 32, 43, 63, 83; 10, 20, 30, 40, 59, 79, 99	−1.71, −2.78, −3.86, −4.93, −6, −7.1, −8.15
3; 9	3, 13, 23, 33, 45, 65, 85; 9, 19, 29, 39, 57, 77, 97	−0.65, −1.06, −1.47, −1.88, −2.3, −2.7, 3.11
4; 8	4, 14, 24, 34, 47, 67, 87; 8, 18, 28, 38, 55, 75, 95	0.65, 1.1, 1.47, 1.88, 2.3, 2.7, 3.11
5; 7	5, 15, 25, 35, 49, 69, 89; 7, 17, 27, 37, 53, 73, 93	1.71, 2.78, 3.86, 4.93, 6, 7.1, 8.15
6	6, 16, 26, 36, 51, 71, 91	2.11, 3.44, 4.77, 6.1, 7.42, 8.75, 10.08
CN2×3		
1; 10	42, 62, 82; 60, 80, 100	−7.06, −8.32, −9.58
2; 9	44, 64, 84; 58, 78, 98	−4.36, −5.14, −6
3; 8	46, 66, 86; 56, 76, 96	0, 0, 0
4; 7	48, 68, 88; 54, 74, 94	4.36, 5.14, 6
5; 6	50, 70, 90; 52, 72, 92	7.06, 8.32, 9.58

**Table 3:** Optimized amplitudes for the PAAS of the two CRA.

Model	Amplitudes
Configuration 1	1.9998 0.3621 1.8963 1.9419 0.2094 1.9184 0.1059 1.6764 1.8317 0.3447 0.9966 0.4440 0.2555 0.1302 0.6474 0.4949 0.4979 0.1406 0.3137 0.5950 0.8243 0.5703 0.1405 0.3697 0.2117 0.2139 0.5985 0.3781 0.3781 0.3781
Configuration 2	0.4151 1.1743 1.2849 1.3340 0.5434 0.5843 1.3178 1.4168 1.1669 1.3718 1.3698 1.1058 1.4535 1.2601 0.7342 1.3838 1.3736 1.1524 1.3929 0.6461 0.8586 1.2891 1.2774 1.1981 1.4290 1.2180 1.4155 1.4239 1.1275 1.2777 0.6157 0.9340 0.4964 0.7554 0.8111 0.4849 0.7463 0.7183 0.9163 0.8420 0.5775 1.0298 0.8356 1.2533 0.5876 0.7050 0.6295 1.0512 0.8737 0.7361 0.5168 0.8362 0.7704 0.8781 0.7116 0.8127 0.5718 1.0242 0.5790 0.8141 0.6353 0.5105 0.5185 0.8109 1.0423 1.0958 0.4863 0.6007 0.5798 0.4462 0.4412 0.5110 0.6597 1.0407 0.8082 0.7466 0.7569 0.5486 0.4373 0.4019 0.5701 0.5093 0.5716 0.8326 1.2098 1.4269 1.1416 1.0053 0.4846 0.6187 0.6801 0.4758 0.5502 0.7493 0.7493 1.3071 1.3102 1.1493 0.4216 0.4216

**Table 4:** Attenuation coefficients of the full system design for the CRA configuration 1 shown in Figure 12.

Element of Antenna	Attenuation Coefficient	Element of Antenna	Attenuation Coefficient
1, 3, 4, 6, 8, 9	−1.86 dB	2, 10	−8.13 dB
5, 7	−8.16 dB	11	−7.74 dB
12, 20	−8.39 dB	13, 19	−9.28 dB
14, 18	−9.34 dB	15, 17	−8.5 dB
16	−7.92 dB	21	−8.15 dB
22, 30	−8.16 dB	23, 29	−8.07 dB
24, 28	−7.99 dB	25, 27	−7.88 dB
26	−7.91 dB		

**Table 5:** Comparison between different works of concentric rings arrays with reduced phase shifters.

	Optimization	Number of Elements	Number of Phase Shifters	Reduction of Phase Shifters (%)	Scanning Range	Feeding Technique	Peak SLL (dB)
Conv. case	No	30	30	0%	±90°(θ)	Uniform	−10
This work: Conf. 1	Yes	30	20	33%	±25°(θ) Scan loss = 0.5 dB	C-BFN	−24 (sim.), −22.5 (Measured)
This work: Conf. 2	Yes	100	60	40%	±25°(θ)	C-BFN	−27.9 (Full-wave)
[18]	Yes	90	60	33%	Not specified	C-BFN	−25 (Array factor)
[21]	Yes	92	87	6%	Not specified	C-BFN	−23 (Array factor)
[28]	Yes	49	Not specif.	0%	Not specified	Uniform	−34 (Array factor)
[29]	Yes	201 142 134	Not specif.	0%	Not specified	Uniform	−29.03 −28 (Array factor) −29.07
[30]	Yes	18 30	Not specif.	0%	Not specified	Uniform	−36 (Array factor) −32.88

## Data Availability

Not applicable.
